# Towards a High-Flux Separation Layer from Hexagonal Lyotropic Liquid Crystals for Thin-Film Composite Membranes

**DOI:** 10.3390/membranes11110842

**Published:** 2021-10-29

**Authors:** Senlin Gu, Bao Yuan, Bo Bai, Xin Tong, Luke A. O’Dell, Dong Wang, Lingxue Kong, Guang Wang

**Affiliations:** 1 Institute of High Energy Physics, Chinese Academy of Sciences, Beijing 100049, China; gusen@deakin.edu.au (S.G.); yuanbao@ihep.ac.cn (B.Y.); baibo@ihep.ac.cn (B.B.); tongxin@ihep.ac.cn (X.T.); 2 Spallation Neutron Source Science Centre, Dongguan 523803, China; 3 Institute for Frontier Materials, Deakin University, Geelong, VIC 3216, Australia; luke.odell@deakin.edu.au; 4 Hubei Key Laboratory of Advanced Textile Materials & Application, Hubei International Scientific and Technological Cooperation Base of Intelligent Textile Materials & Application, Wuhan Texture University, Wuhan 430200, China; Wangdon08@126.com

**Keywords:** hexagonal lyotropic liquid crystal, hydrophobic/hydrophilic substrates, thin-film composite membranes

## Abstract

Hexagonal lyotropic liquid crystals (HLLC) with uniform pore size in the range of 1~5 nm are highly sought after as promising active separation layers of thin-film composite (TFC) membranes, which have been confirmed to be efficient for water purification. The potential interaction between an amphiphile-based HLLC layer and the substrate surface, however, has not been fully explored. In this research, hydrophilic and hydrophobic microporous polyvinylidene fluoride (PVDF) substrates were chosen, respectively, to prepare TFC membranes with the active layers templated from HLLC, consisting of dodecyl trimethylammonium bromide, water, and a mixture of poly (ethylene glycol) diacrylate and 2-hydroxyethyl methacrylate. The pore size of the active layer was found to decrease by about 1.6 Å compared to that of the free-standing HLLC after polymerization, but no significant difference was observable by using either hydrophilic or hydrophobic substrates (26.9 Å vs. 27.1 Å). The water flux of the TFC membrane with the hydrophobic substrate, however, was higher than that with the hydrophilic one. A further investigation confirmed that the increase in water flux originated from a much higher porosity was due to the synergistic effect of the hydrophilic HLLC nanoporous material and the hydrophobic substrate.

## 1. Introduction

The drastic growth of the economy and population has led to a wide scarcity of water resources around the world [1]. Membrane technologies have been playing important roles in obtaining fresh water from seawater and brackish water. Thin-film composite (TFC) membranes consisting of an active separation layer, a microporous support, and a polyester non-woven backing layer have been regarded as the state-of-the-art design due to their high permselectivity, chemical stability, and compaction resistance [1]. The middle microporous support layer enables the active separation layer to withstand high-pressure compression. The active separation layer, on the other hand, mainly contributes to the nanofiltration performance. The pore size, porosity, and continuity of nanopores are, therefore, key parameters for the active layer to perform well [2], while its thickness needs to be minimized to decrease the resistance for a high permeability. 

An extensively employed material serving as the active layer is aromatic cross-linked polyamide (PA), whose great rejection performance is due to the free-volume holes among polymeric chains, typically 0.2–0.29 nm [3]. However, this contributes to the main resistance to water transport and, therefore, the water flux of TFC membranes with PA applied as the active layer is very low [4]. Alternative materials to replace PA, including polyelectrolytes [5], nanocomposites [6,7,8,9,10], and ceramics [11,12,13], suffer from limitations in either mechanical strength, uniformity of pore size, or cost-effectiveness.

Lyotropic liquid crystals (LLC) are a promising option to serve as templates for the active separation layers because of the resulting inherently uniform and controllable pore size, ranging from 0.2–5 nm [14,15,16,17,18,19], with which the water treatment can be performed based on molecular size exclusion of hydrated salt ions with minimal external forces required [20]. TFC with an active layer of uniform pore size at 1 nm possesses a water permeability of 2 Lm^−2^h^−1^bar^−1^μm and exhibits an effective inorganic salt rejection and a molecular weight cutoff of about 300 Da for organic molecules [14]. Gin et al. reported an active separation layer templated from LLC with an effective pore size of 0.29–0.45 nm, possessing a high salt rejection rate and a water flux similar to that of commercially available reverse osmosis membranes (0.086 vs. 0.080 Lm^−2^h^−1^bar^−1^μm) in dead-end filtration tests [17]. The templating method for HLLC to serve as the active layer is more straightforward because the continuity of the straight nanopores can be enhanced by using appropriate strategies [21,22,23,24] and a significantly high flux can be achieved accordingly. We previously fabricated a robust nanoporous material by using the template method of HLLC [25]. The material presents huge potential for serving as the active separation layer, but the fabrication of TFC membranes by using this amphiphile-based HLLC system has not yet been performed.

The microporous substrate also plays an important role in not only mechanically supporting the active layer, but also exerting great effects on its structural control and filtering performance [26,27,28]. It has been found that the porous substrate could be used as the storage reservoir of monomers for interfacial polymerization. The surface properties and porous structure of the substrate can greatly influence the diffusion of the monomers and, thus, the location of the water–oil interface. As a consequence, these effects are highly related to, or even dominate, the thickness and polymeric structure of the active layer and the filtration performance of TFC membranes. It has been confirmed that a support layer with higher porosity, stronger hydrophilicity, and rougher surface could impart a TFC membrane with a much higher water flux [29,30,31]. Specifically, the key role of the hydrophilicity of substrates in adjusting the water flux of TFC membranes has been proved from various systems [32,33,34]. Therefore, it is particularly important to examine the effects of hydrophilicity of the substrate on the preparation of TFC membranes when HLLC is applied as the active layer due to the presence of amphiphiles. This, in turn, attracts interest in how an amphiphile-directed system interacts with the substrates possessing various surface properties and the subsequent structural retention, pore size, and filtration performance of the prepared TFC membranes. However, little information about this is available. 

Herein, an HLLC system composed of dodecyl trimethylammonium bromide (DTAB), water, poly (ethylene glycol) diacrylate (PEGDA), and 2-hydroxyethyl methacrylate (HEMA) was used to fabricate TFC membranes on PVDF substrates with opposite surface properties. The variations in the nanostructure, interfacial morphology between the active layer and the substrate, and water flux were examined. The retention of the HLLC structure with and without substrates after polymerization was verified by an X-ray diffractometer, nitrogen adsorption analyzer, polarized optical microscope and scanning electron microscope, respectively. The internal porosity was found to be responsible for the water flux of the prepared TFC membranes and a high flux could be achieved by tuning the hydrophilicity of the substrate.

## 2. Materials and Methods

### 2.1. Materials

Chemicals were all purchased from Sigma-Aldrich Company (St Louis, MO, USA). PEGDA, HEMA, and 2-hydroxy-2-methylpropiophenone (HMPP, photo-initiator) were stored at 4 °C. DTAB was stored in the desiccator. All chemicals were used as received. PVDF substrates with various surface properties were purchased from Haining Yibo Guolv Company (Haining, China). The thickness, pore size, and diameter of the PVDF substrates were 0.090 ± 0.001 mm, 0.45 ± 0.02 μm, and 5 cm, respectively. The hydrophilic PVDF substrate surface treated by the chemical soaking method possessed a large number of hydrophilic groups, such as carboxyl and hydroxyl groups, compared to that of the hydrophobic one.

Sample preparation: a mixture of PEGDA and HEMA was added into the binary system of DTAB and H_2_O (DTAB/ H_2_O /PEGDA/HEMA = 49.4/27.3/4.5/18.8, *v*/*v*). HMPP (0.5 wt %) was added into the ternary system for photo-polymerization. The mixture was stored in a small vial sealed with para-film. The vial was put into a water bath at 40 °C with magnetic stirring until a homogenous transparent liquid crystal sample was observed (at least 12 h). Then, the mixture was kept at ambient temperature for 12 h prior to being further processed into membranes with/without PVDF substrates. 

**Figure 1:**
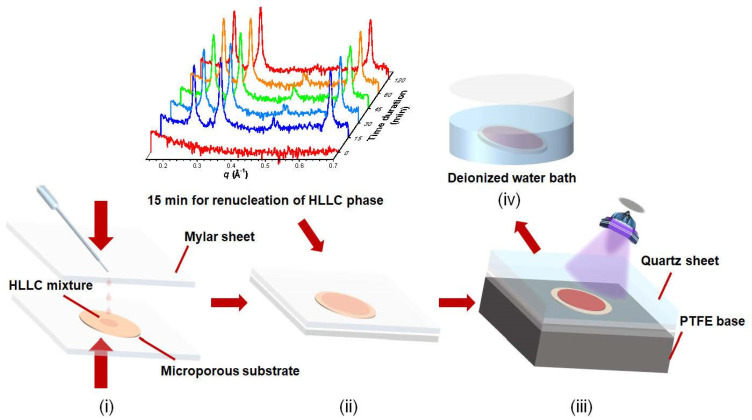
Schematic showing the hot-pressing process for the fabrication of TFC membranes. (**i**) hot pressing, (**ii**) cooling, (**iii**) UV curing and (**iv**) surfactant removal.

Fabrication of TFC membranes: The TFC membranes were fabricated by using a hot-pressing method (Figure 1)**.** Firstly, 15 drops of the HLLC mixture, after being heated to melting point (~0.26 g), were placed on a piece of microporous support. The mixture, together with microporous support, was sandwiched between Mylar sheets. The entire assembly was then placed between two smooth iron plates for pre-heating at 50 °C for 2 min, followed by pressing with 1 MPa for 5 min to infuse the mixture through the support film. Secondly, the assembly was cooled at ambient temperature (25 °C) for 15 min for renucleation of the HLLC phase. Thirdly, the assembly was UV-cured for 0.5 h. The membranes were finally immersed into deionized water to remove the surfactant molecules and facilitate peeling the TFC membranes off from the Mylar sheets. 

### 2.2. Characterizations

X-ray diffraction (XRD): A lab-sourced Rigaku Smartlab X-ray instrument (Japan) with a general Bragg–Brentano focusing mode monochromatized with Cu K*α* (*λ* = 1.54 Å) radiation was applied to initially distinguish the HLLC structure and examine the variation in *d*-spacing. *θ*/2*θ* scans from 0.5° to 10° with a speed of 0.5°/min and a step of 0.02° were carried out at room temperature (25 °C). The free-standing HLLC mixture was characterized on a round frosted glass tank, while an iron platform was used for that of the TFC membranes. All XRD patterns were corrected and background subtracted.

Polarized optical microscopy (POM): The HLLC nanostructure before and after polymerization was verified by POM (ZEISS Axiolab 5, Hamburg, Germany) equipped with a digital camera. The HLLC mixture was diluted by acetone and the hexagonal phase was captured during the volatilization of acetone before performing polymerization. A cover glass was required to avoid the water evaporation from the HLLC mixture during photo-polymerization and then the micrographs of the polymerized free-standing HLLC mixture were captured. 

Dehydration methods: The supercritical CO_2_ method was used to dry membranes made of the free-standing HLLC mixture. The samples were first dehydrated by gradient ethanol with an increasing ethanol ratio (deionized water/ethanol, *w*/*w*) of 2:1, 1:1, 1:2, 0:2 for 3 h each [35]. Then, ethanol was replaced by liquid CO_2_, followed by dehydration under the supercritical point of CO_2_ (304 K, 7.39 MPa) for 15 min. The TFC samples were dried in a fuming cupboard.

A scanning electron microscope (SEM) was used to observe the cross-sectional surface of the free-standing HLLC mixture and the TFC membranes. The free-standing HLLC membranes were observed by using a Hitachi S-4800 (Japan) field emission scanning electron microscope. The samples were fractured in liquid nitrogen and the cross-sectional sample surface was coated with a thin layer of gold before the SEM observations. The cross-sectional surface of the TFC membrane was fast cut by a surgical blade before gold coating and then observed using a ZEISS Gemini-300 field emission scanning electron microscope (Germany). 

N_2_ physisorption measurements were conducted for the surface area and the pore size of the free-standing HLLC mixture membranes after polymerization (TriStar II 3020, America). N_2_ adsorption and desorption isotherms were recorded at −196 °C. The specific surface area and pore size distribution curves were determined by the Brunauer–Emmett–Teller (BET) method and the Barrett–Joyner–Halenda (BJH) method, respectively. Samples were degassed in a vacuum for 4 h at 200 °C before measurements.

The flux test was carried out via a crossflow filtration apparatus (SF-SA, Saifei Membrane Separation Technology Co., Hangzhou, China) with an effective filtration area of 7.07 cm^2^ at room temperature. The water flux was determined under a pressure of 5 bar and a crossflow flux of 35 L/h. A deionized water-wetted membrane was fixed in the cell of the apparatus. Before the test, the membrane was pre-pressed with an inlet pressure of 5 bar for 5 min. The water flux was calculated by recording the volume of permeate after the first 30 min. Water flux (J) was determined by Equation (1) [36]:

(1) $$J=\frac{\Delta w}{\rho A\Delta t}$$

where Δ*w* is the weight increase of permeate during the filtration test, Δ*t* is the time duration of the test, *A* is the effective separation area of the cell, and *ρ* is the density of the permeate (1 kg·L^−1^).

Contact angle tests were carried out by using a contact angle measuring device (KRUSS DSA30S, Germany). The substrates were cut into 2 cm^2^ sheets and fixed on a silicon wafer using Kapton tape. A droplet of deionized water (5 μL) was dropped onto the surface of the samples. The images of the drop were recorded at 1 s and 60 s, respectively.

The water absorption was measured according to the dry–wet weight. The wet TFC was dried in a fume cupboard, followed by measuring the weight of the dried TFC, which was immersed in deionized water for 24 h again. The weight of the wetted membrane was measured again after removing the excess water on its surface by using filter papers. The water absorption (ε (%)) can be calculated using Equation (2):

(2) $$\varepsilon \left(\%\right)=\frac{\left(W_{w}-W_{D}\right)}{W_{D}}\times 100\%$$

where *w_w_* is the weight of the wetted TFC membranes and *w_D_* is that of the dried ones.

## 3. Results and Discussion

### 3.1. Structure Characterization for Free-Standing HLLC Template Membrane

**Figure 2:**
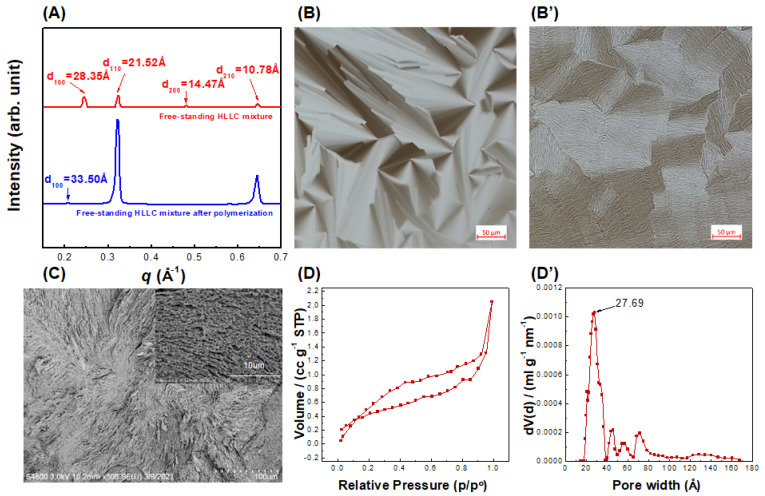
High-fidelity retention of the nanostructure of free-standing HLLC mixture and its characterizations. (**A**) XRD profiles before and after polymerization, polarized optical micrographs (**B**) before and (**B’**) after polymerization, (**C**) scanning electron micrographs after polymerization, (**D**) N_2_ adsorption and desorption isotherms, and (**D’**) pore size distribution curve after polymerization.

A template-sacrificial method was carried out to fabricate the HLLC template’s nanoporous materials. The cross-linkable monomers were dissolved in the hydrophilic areas of the formed HLLC phase, and then the monomers were free-radically polymerized by UV curing to retain the template structures, followed by surfactant removal to achieve a nanoporous structure with cylindrical pores. In this process, the nanostructural preservation of the free-standing HLLC mixture during UV curing is important for the fabrication of TFC membranes. The structure of the free-standing HLLC mixture before and after polymerization was verified by XRD, POM, SEM, and N_2_ physisorption measurements. The *d*-spacing ratios of 1, 1/3^1/2^, 1/4^1/2^, and 1/7^1/2^ for *d*_100_, *d*_110_, *d*_200_, and *d*_210_ in the XRD curves correspond to an HLLC phase (Figure 2A) [37]. The *d*_100_ value of the free-standing HLLC mixture increased by 5.15 Å after polymerization. The diameter within the hexagonal column can be evaluated via *d*_100_, which can be used to deduce the pore size of the hexagonal mesophase [38,39]. It was calculated that the column diameters of the free-standing HLLC mixture before and after polymerization were 24.17 ± 0.02 Å and 28.56 ± 0.04 Å, respectively. The structural retention of the free-standing HLLC mixture before and after polymerization was further verified by POM. A typical focal conic texture representative of an HLLC phase before polymerization was observed (Figure 2B). This birefringence texture was retained to a large degree after photo-polymerization, as shown in Figure 2B’. The well-retained optical texture after polymerization demonstrates that the parent template structure was well preserved during photo-polymerization. 

SEM was used to directly observe the structural morphology of the polymerized free-standing HLLC mixture after surfactant removal (Figure 2C). The isotropic pore channel structure was observable on the cross-sectional surface of the polymerized sample. It should be noted that this isotropic morphology was caused by the isotropic distribution of the hexagonal LLC domains. It was interesting to find that an anisotropic rod-like morphology could be observed after zooming in (Figure 2C inset), which has been proved to be induced by the HLLC parent template [40]. However, the dimension of these rod-like structures was about 100 times larger than the *d*-spacing of the HLLC system, which was caused by the swelling and aggregation of nanostructures during sample preparation (supercritical CO_2_ drying and freezing fractured in liquid nitrogen). As the water must be removed before the SEM experiments and the sample structure could inevitably change during the sample preparation, this rod-like structure is evidence of structural retention after polymerization [40]. 

To further study the pore size distribution of the HLLC mixture after polymerization, N_2_ physisorption measurements for supercritical CO_2_ dried HLLC mixture were carried out (Figure 2D). This isotherm was classified as a type IV isotherm and H3 hysteresis loop, corresponding to typical mesoporous materials. A most frequent pore diameter of approximately 27.69 Å was exhibited, as shown in Figure 2D’. Some larger pore sizes were due to the defects created during the drying process. The pore size from the N_2_ physisorption measurement (27.69 Å) was in strong agreement with that from the X-ray diffraction (28.56 Å), confirming that a mesoporous nanomaterial from the free-standing HLLC template with a uniform pore size of ~28 Å was achieved.

### 3.2. Structure and Property Characterization for HLLC Template-Based Thin-Film Composite

**Figure 3:**
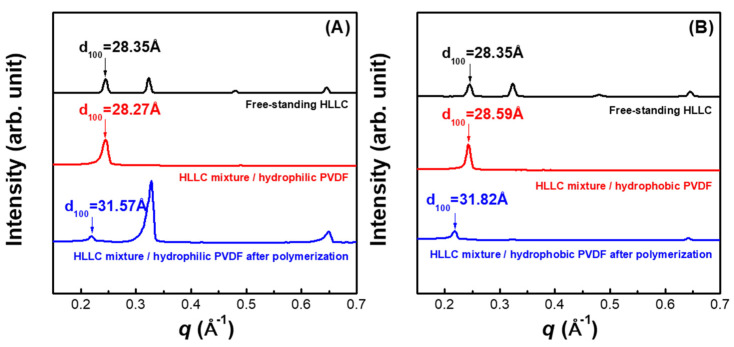
XRD profiles of TFC with (**A**) hydrophilic and (**B**) hydrophobic PVDF.

Figure 3 shows the XRD profiles for the TFC membranes with hydrophilic and hydrophobic PVDF substrates before and after polymerization. It can be observed that the HLLC phases formed on both hydrophilic and hydrophobic PVDF substrates and the structural dimensions of the HLLC mixture were identical. Polymerization of the HLLC mixture on hydrophilic and hydrophobic PVDF membranes increased *d*_100_ by 3.30 Å and 3.23 Å, respectively. The increase in the *d*_100_ of the HLLC mixture on substrates after polymerization was lower than that of the free-standing HLLC mixture after polymerization (~5 Å), which could be attributed to the substrate effects during the polymerization. Table 1 shows the dimensional evolution of the nanostructures for all the samples before and after polymerization. It was observed that the column diameters were similar for the free-standing HLLC mixture (28.35 ± 0.06 Å), HLLC mixture/hydrophilic PVDF (28.27 ± 0.07 Å), and HLLC mixture/hydrophobic PVDF (28.59 ± 0.07 Å) before polymerization, suggesting the surfaces of both hydrophilic and hydrophobic PVDF had little effect on the HLLC phase formation. Intriguingly, the polymerized active layers on hydrophilic and hydrophobic PVDF possessed similar column diameters of 26.91 ± 0.01 Å and 27.12 ± 0.01 Å, respectively, which were smaller than that of the polymerized free-standing HLLC mixture by ~1.6 Å. These results suggest that the substrates could influence the structural evolution of the HLLC parent template during polymerization.

**Figure 4:**
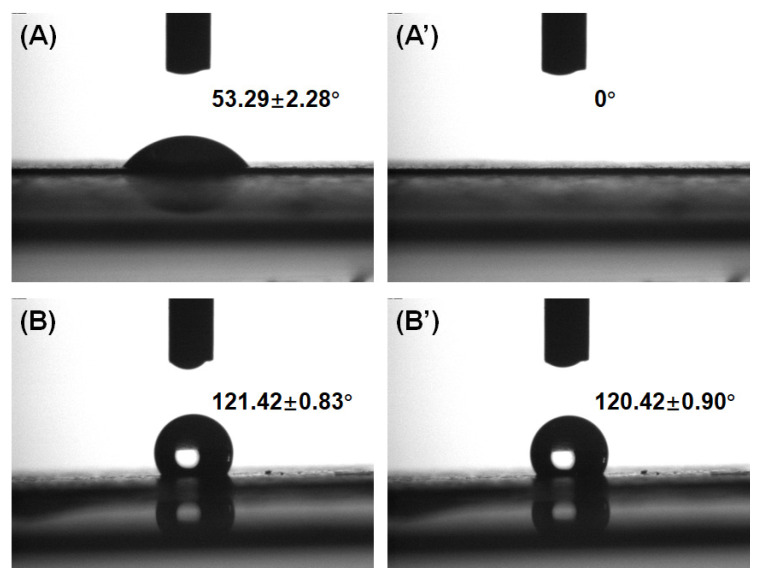
Contact angle results for (**A**) hydrophilic and (**B**) hydrophobic PVDF after 1 s; (**A’**) hydrophilic and (**B’**) hydrophobic PVDF after 1 min.

**Figure 5:**
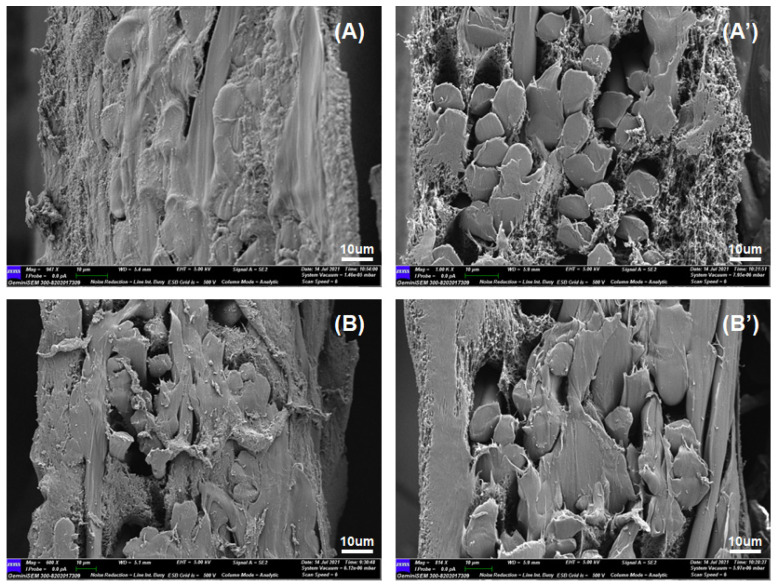
Scanning electron micrographs of HLLC mixture on (**A**) hydrophilic and (**B**) hydrophobic PVDF after polymerization; (**A’**,**B’**) are primitive hydrophilic and hydrophobic PVDF substrates, respectively.

Water filtration tests of the microporous substrates and TFC membranes with various microporous substrates were performed. The flux data for each sample are shown in Table 2. It was interesting to find that the water flux of the TFC membrane with the hydrophilic PVDF substrate was smaller than that with the hydrophobic one, while the hydrophilic substrate possessed a higher water flux than the hydrophobic one (the contact angles for the hydrophilic and hydrophobic substrates were 53.29 ± 2.28° and 121.42 ± 0.83°, respectively, as shown in Figure 4). The cross-sectional surface of the TFC membranes was observed by SEM (Figure 5). It was observed that the two primitive PVDF substrates showed a similar cross-sectional morphology, while the porosity of the TFC membrane with the hydrophobic PVDF was higher than that with the hydrophilic one. 

### 3.3. Discussion

In general, the pore size, surface hydrophilicity, thickness, and porosity determined the water flux of the TFC membranes. The pore size of the HLLC nanoporous active layer dominated the water flux of the TFC membranes. As there was no obvious difference in the HLLC structure after polymerization on both hydrophilic and hydrophobic PVDF substrates, the pore size of the active layer, therefore, had insignificant effects on the water flux of the TFC membranes. Since the HLLC mixture was infused into the substrate, the surface properties of the TFC membranes were, therefore, determined by the substrate to some extent. It is rational to expect that TFC membranes would exhibit higher water flux with a hydrophilic surface than with a hydrophobic one, but this is not in agreement with the results of this study, where that the higher water flux by using the hydrophobic substrate could not be ascribed to the hydrophilicity. Moreover, the TFC membrane with the hydrophobic PVDF substrate (137 ± 1 μm) was much thicker than that with the hydrophilic one (100 ± 1 μm), which also contradicts the water flux results. The water absorption of the hydrophilic PVDF substrate was 68.6 ± 0.2%, while it was only 5.6 ± 1.4% for the hydrophobic one. The water absorptions of the TFC membranes with the hydrophilic and hydrophobic PVDF substrate, however, were similar, at 42.5 ± 0.8% and 41.1 ± 0.6%, respectively. Rationally, the water absorption of the TFC membrane with the hydrophilic PVDF substrate should have been much higher than that with the hydrophobic one if the porosities of TFC membranes with the hydrophilic and hydrophobic substrates were the same. Therefore, it can be concluded that the higher porosity of the TFC membrane with the hydrophobic PVDF substrate contributed to the increase in water absorption and enabled it to be comparable to that of the TFC membrane with the hydrophilic PVDF substrate. Consequently, the higher water flux of the TFC membrane with the hydrophobic PVDF substrate can only be explained by the higher porosity achieved by the interaction between the hydrophilic HLLC nanoporous materials and the hydrophobic PVDF substrate.

## 4. Conclusions

We successfully fabricated a new high-flux TFC membrane with the active layer templated from hexagonal lyotropic liquid crystals and the hydrophobic PVDF selected as the substrate layer. A decrease of ~1.6 Å in pore size was found after UV curing the HLLC mixture on the substrates in comparison with the free-standing HLLC mixture, while a similar pore size of the active layer was found for TFC membranes with hydrophilic and hydrophobic PVDF substrates, respectively. The porosity of TFC membranes can be tailored by adjusting the hydrophilicity of the substrates, which leads to a TFC membrane with high water flux by combining the hydrophilic HLLC nanoporous material and the hydrophobic PVDF substrate. Such a method of combining the HLLC nanomaterials and substrates with various surface properties paves a new road to fabricate the TFC membrane with high water flux and the industrial viability of the membrane fabrication process makes it possible for large-scale production. We expect that additional developments can be achieved by optimizing formulation and processing to provide new solutions for the trade-off effect between selectivity and permeability in water filtration. 

## Figures and Tables

**Table 1:** Dimensional evolution of structure for *d*_100_ (a) before and (b) after polymerization and column diameter (c) before and (d) after polymerization.

Samples	a (Å)	b (Å)	c (Å)	d (Å)
Free-standing HLLC mixture	28.3 ± 0.06	33.5 ± 0.06	24.1 ± 0.02	28.6 ± 0.04
HLLC mixture/hydrophilic PVDF	28.3 ± 0.07	31.6 ± 0.03	24.1 ± 0.03	26.9 ± 0.01
HLLC mixture/hydrophobic PVDF	28.6 ± 0.07	31.8 ± 0.03	24.4 ± 0.02	27.1 ± 0.01

**Table 2:** Water flux for microporous substrates and TFC membranes.

Samples	Pressure (bar)	Cross Flow Flux (L/h)	Flux (L/m^2^⸳h)
Hydrophilic PVDF (blank)	5	35	17,822 ± 892
Hydrophobic PVDF (blank)	5	35	15,191 ± 773
HLLC mixture/hydrophilic PVDF after polymerization	5	35	517 ± 53
HLLC mixture/hydrophobic PVDF after polymerization	5	35	720 ± 60

## Data Availability

Not applicable.
